# Estimating geographic access to healthcare facilities in Sub-Saharan Africa by Degree of Urbanisation

**DOI:** 10.1016/j.apgeog.2023.103118

**Published:** 2023-11

**Authors:** Pietro Florio, Sergio Freire, Michele Melchiorri

**Affiliations:** European Commission, Joint Research Centre, Ispra, Italy

**Keywords:** Urbanization, GHSL, SDG, Cities, Rural areas, Health planning

## Abstract

Measuring rates of coverage and spatial access to healthcare services is essential to inform policies for development. These rates tend to reflect the urban-rural divide, typically with urban areas experiencing higher accessibility than rural ones. Especially in Sub-Saharan Africa (SSA), a region experiencing high disease burden amid fast urbanisation and population growth. However, such assessment has been hindered by a lack of updated and comparable geospatial data on urbanisation and health facilities. In this study, we apply the UN-endorsed Degree of Urbanisation (DoU or DEGURBA) method to investigate how geographic access to healthcare facilities varies across the urban-rural continuum in SSA as a whole and in each country, for circa 2020. Results show that geographic access is overall highest in *cities* and *peri-urban areas*, where more than 95% of inhabitants live within 30 min from the nearest HCF, with this share decreasing to 80–90% in *towns*. This share is lowest in *villages* and *dispersed rural areas* (65%), with about 10–15% of population more than 3 h away from any health post. Challenges in geographic access seem mostly determined by high travel impedance, since overall spatial densities of HCF are comparable to European levels.

## Highlights

• The Degree of Urbanisation enables monitoring the urban-rural access to resources and services across boundaries worldwide.
• This work characterizes distribution and accessibility of Health Care Facilities (HCF) by Degree of Urbanisation (DoU).
• The accessibility to HCF at given travel time bins is shown by HCF tier of specialisation and share of covered population.
• Access to HCF varies significantly across countries, and within countries by DoU territorial class (Fig. 7).
• HCF are widespread in Sub-Saharan Africa but the inadequate infrastructure makes them inaccessible, notably in rural areas.

## 1 Introduction

According to most population statistics, African cities are outpacing the rest of the world in growth. In spite of decreasing birth rates, the medium term effect of population structures and living conditions result in a highly populated region, with striking implications for the coming decades. Out of the 100 most populous cities in the world, 38 will be in Africa in 2100 (Hoornweg & Pope, 2017). Nevertheless, population dynamics in rural areas and sparse settlements constitute a significant demographic component of national population structures.

Sub-Saharan Africa (SSA) is projected to become the most populous world region in the late 2060s (among the eight world regions identified by the United Nations). Doubling its inhabitants between 2022 and 2050 (United Nations, Department of Economic and Social Affairs, Population Division, 2022), SSA will account for 35% of the global population by 2100 (from 13% in 2017) (Ezeh et al., 2020). Urbanisation in SSA is a sizable process both in terms of demographic and land urbanisation components (Linard et al., 2013). In terms of demographic urbanisation, most countries in SSA have doubled their urban population between 1990 and 2015 (European Commission, Joint Research Centre, 2019); concurrently, estimates derived from Earth Observation identified that, over the same period, urban expansion exceeded 30,000 km^2^ of newly built-up areas in cities in the African region (Angel, 2011; Melchiorri et al., 2018). Studies on urban expansion indicate that adequate spatial planning is needed to manage the scale, extent and speed of urban expansion in the region.

Knowledge of urbanisation and human settlement patterns are determinant for assessing and planning the provision of basic services supporting development and well-being, such as healthcare. In general, sub-Saharan Africa continues to experience serious shortcomings regarding the provision of adequate and accessible healthcare. Overall, Africa hosts less than three medical doctors per 10 thousand inhabitants (World Health Organization, 2022), while suffering a high disease burden, including from health issues which could be substantially controlled with an appropriate prevention. In 2019, infectious diseases caused more than 400 deaths per 100 thousand inhabitants in SSA, compared to 26 and 18 in Europe and North-America respectively (Global Burden of Disease Collaborative Network, 2021). In 2020, SSA shouldered about 93% of malaria deaths globally (World Health Organization, 2021) and in 2017, 75% of deaths and 65% of new infections related to HIV occurred in SSA (Dwyer-Lindgren et al., 2019). This is increasingly dramatic considering that almost 90% of population is SSA does not access any health insurance (Amu et al., 2022), and one in eight people lives more than 1 h away from the nearest health centre (Falchetta et al., 2020). More recent accessibility statistics refined these estimates (Hierink et al., 2022).

Despite the political commitment expressed in the Abuja declaration of 2001, which pledged to allocate 15% of the public expenditure to healthcare, in 2014 the average annual public expenditure on health in Africa was 10% of the total public spending, with few to no countries meeting the 15% goal (World Health Organisation, 2016). Increasing access to healthcare is a priority included in the United Nations Sustainable Development Goals (SDGs). Among other indicators, the United Nations (UN) assess the coverage of essential health services (SDG 3.8), considering the number of beds per inhabitant (United Nations Human Settlements Programme (UN-Habitat) & United Nations Statistics Division (UNSD), 2023).

One well established baseline analysis to inform such development policies is the estimation of geographic access to healthcare facilities (HCF), as a measure of potential physical access by the population of a country or region (Kotavaara et al., 2021; McGrail & Humphreys, 2014). However, the lack of detailed and up-to-date spatial data and health information have limited the feasibility and accuracy of such analyses (Pu et al., 2020), especially in large, developing world regions such as SSA. Narrowing this gap, the World Health Organisation (WHO) has recently created a geospatial centre for health (WHO GIS Centre for Health)^1^ working towards tracing the name, location and type of HCF worldwide by 2027,^2^ with such data for SSA already being compiled.

Research already identified a positive correlation between human development and access to healthcare (Nuhu et al., 2018). Income is not the only factor that explains higher access to healthcare; education is also crucial. Access to basic services, such as electricity, water and sanitation has important health outcomes (Parienté, 2017), binding SDGs together into inter-connected challenges. The territorial distribution of points of interest, such as services, follows several optimization functions related to different siting logics. Trade-offs sometimes arise when efficiency and coverage come into play. Oftentimes, the urban/rural divide manifests as unequal provision and access to services.

In SSA, a few studies examined the differences in urban/rural availability of healthcare to specific target groups, like pregnant women (Samuel et al., 2021), adult population (Menashe-Oren & Stecklov, 2018), or respondents to a satisfaction survey about healthcare in Ghana (Yaya et al., 2017). While a recent study looked into measures of geographic access to healthcare (Hierink et al., 2022), how that accessibility varies geographically across the urban/rural continuum remained unknown. One obvious obstacle has been that national definitions of ‘urban’ and ‘rural’ areas, when available, often lack a statistical and geospatial basis to support analysis, and their wide concepts and criteria make inter-comparison of results untenable (Dijkstra et al., 2020).

The UN-endorsed Degree of Urbanisation^3^ method (DoU) overcomes such limitations by providing a methodology for the delineation of cities, as well as urban and rural areas, for international and regional statistical comparison purposes, supporting the collection of harmonised indicators requested by global agendas (e.g. SDG, New Urban Agenda). The globally harmonised method was explicitly designed to monitor access to services and infrastructure in areas with different population sizes and densities, being more objective as it does not factor the presence of facilities in the definition. In addition, at its second level, the DoU delineates seven classes of settlements, enabling analysis beyond the traditional urban/rural divide and into the gradient of settlement size and density.

In this study, we employ the Degree of Urbanisation method to investigate how geographic access to healthcare facilities varies across the urban-rural continuum in SSA as a whole region and in each country, for circa 2020. The novelty of this approach resides in the adoption of a definition of urban and rural areas that gathers an international consensus and allows for comparisons across countries. We start by classifying all level-2 administrative areas and we geographically match the resulting classification with existing datasets collecting information about healthcare in SSA: we use the latest data on healthcare facilities to assess their distribution and density by each of seven settlement typologies (second level DoU). Then, we look at the relative distribution of healthcare facility tiers by settlement typology and compute the shares of population with geographic access to healthcare facilities as a function of travel time. For discussion, we also quantify the amount of people by classes of travel time to the nearest urban centre.

In particular, this work's objectives include exploring the relation between the degree of urbanisation of level-2 administrative areas in SSA and: a) the quantity of healthcare facilities within the territory; b) the quality and integration of HCF within the territory; c) the geographic accessibility of HCF within the territory.

## 2 Materials and methods

For the purposes of this work, Sub-Saharan Africa reflects the definition of the World Bank, and the list of 48 countries it comprises,^4^ with the addition of Djibouti: Figure S.4 includes the comprehensive list of all countries in our definition of SSA.

### 2.1 The classification of settlements according to the Degree of Urbanisation

**Fig. 1:**
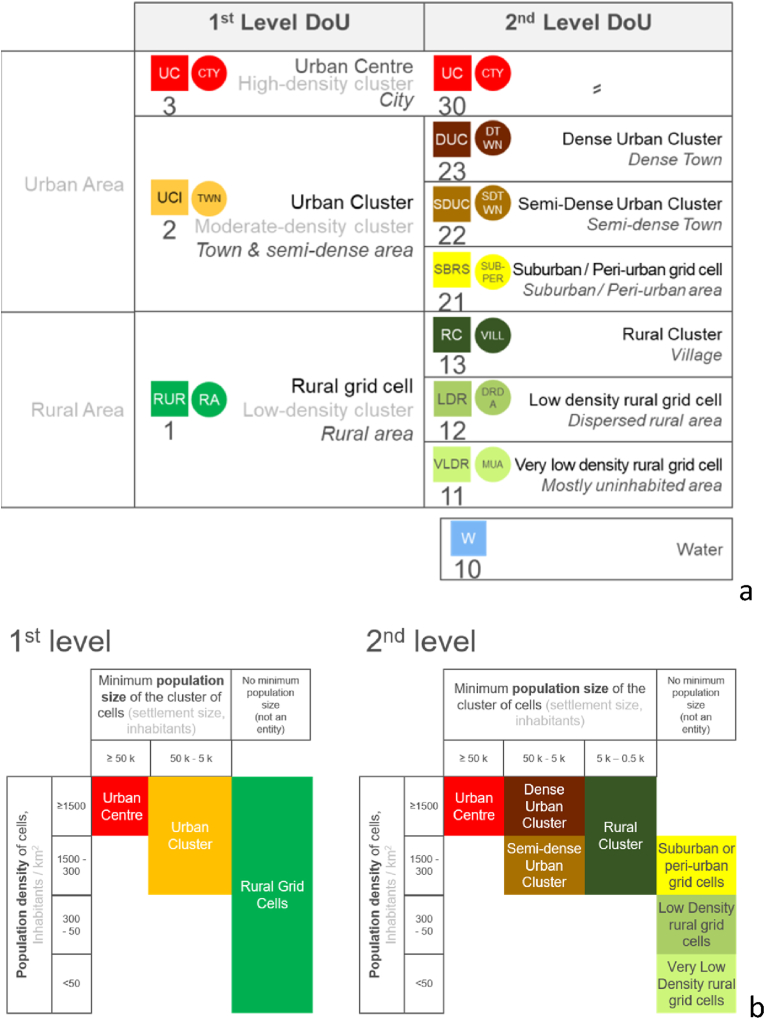
(a) The Degree of Urbanisation classification scheme, with grid terminology (black, straight), technical terminology (grey) and administrative terminology for territorial units (black, italic); numbers correspond to the encoded value in the raster grid file (GHS-SMOD); squares contain the abbreviations of the grid terminology (GHS-SMOD), rounds the ones of the administrative terminology (GHS-DUC). (b) Population size and density thresholds defining the Degree of Urbanisation classes at grid stage.

The classification of settlements relies on the Degree of Urbanisation (DoU), a harmonised method designed to perform such classification worldwide, for international statistical comparisons, based on population density and size (Dijkstra et al., 2020). The method, endorsed by the UN Statistical Commission in 2020 (UN. Statistical Commission, 2020), captures the full spectrum of settlements from the hamlet to the mega-city and can be applied to any population census. It consists of two levels: the first and main level divides a territory into three classes: *cities, towns and semi-dense areas*, and *rural areas. Cities* and *towns* constitute urban areas. A second level provides more detail, by splitting *towns and semi-dense areas* into *towns* and *suburban or peri-urban areas* and splitting rural areas into *villages, dispersed rural areas* and *mostly uninhabited areas*. The Degree of Urbanisation allows carrying out the classification of pixels on a grid of population density with fixed resolution of 1 km; applied on an equal area projection that preserves surface proportions, this is the basis for the classification of overlying territorial units (Fig. 1a). The population size and density thresholds defining the different classes on a grid (Fig. 1b). For instance, *urban centres* are patches of grid cells, with a minimum density of 1500 inhabitants per km^2^ each, exceeding altogether 50 thousand inhabitants.

The Global Human Settlement Layer features a worldwide application of the Degree of Urbanisation method (Melchiorri et al., 2018), based on the global grids of resident population GHS-POP (Schiavina, Marcello, Freire, Sergio, et al., 2022). GHS-POP grids are especially suited to assess population's accessibility to facilities and services in SSA, because while departing from best available census data, their production includes the critical revision of census units deemed “unpopulated” (based on counter-evidence from satellite imagery) and enhanced representation of settlements along coastlines (Freire et al., 2018). Other global population datasets exist, but as all products from the GHS suite are based on GHS-POP, it constitutes an assumption of this research work, for consistency and coherence.

The grid map of the Degree of Urbanisation is openly accessible online as a raster layer under the “Settlement Model” denomination, GHS-SMOD (Schiavina et al., 2022, Schiavina et al., 2022, Schiavina et al., 2022). The global administrative subdivisions in the GADM map (GADM Database of Global Administrative Areas, Version 3.6, 2018) inherit their classification from such worldwide grid, to constitute the “Degree of Urbanisation Classification” of administrative units, GHS-DUC (Schiavina et al., 2022, Schiavina et al., 2022, Schiavina et al., 2022). Both mentioned datasets cover the SSA region, which can be extracted as subset. The finest administrative subdivision covering the whole territory of SSA comprehensively is GADM level 2, which provides the territorial units for classification.

### 2.2 Data integration

**Fig. 2:**
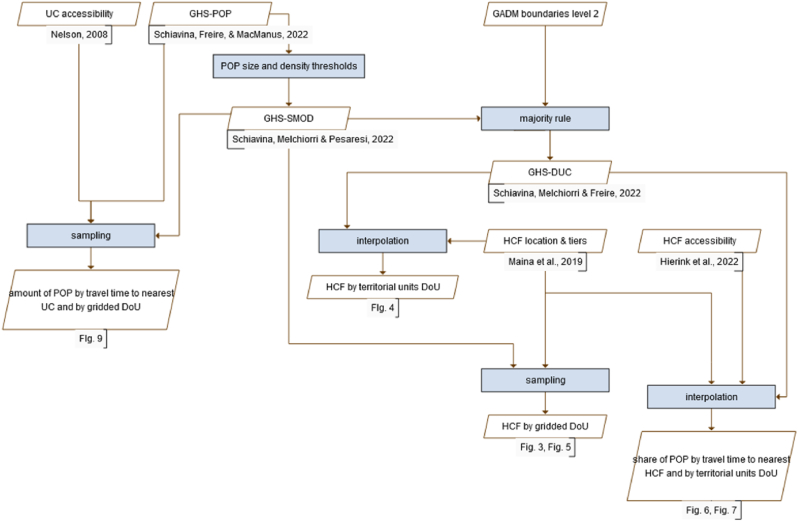
Workflow implemented in this research work.

This study combines four main datasets: one about the urban/rural classification of the territory, two related to healthcare facilities, and one detailing the geographic accessibility of urban centres (Fig. 2). The urban/rural classification is constituted by a grid-based raster map extracted from the global GHS-SMOD, with pixel values corresponding to the DoU categories (see Fig. 1a) and a vector-based polygonal map of administrative areas in SSA issued from the global GHS-DUC (see paragraph 2.1), featuring the DoU class as an attribute in the attribute table. The healthcare datasets contain the location and tier of specialisation of the facilities (Maina et al., 2019) and accessibility to them in terms of travel time (Hierink et al., 2022). The geographic accessibility of urban centres is published as a raster map that encodes the travel time to the nearest urban centre from each pixel, based on a different model (Nelson, 2008). The geospatial location and the unique identifiers of administrative subdivisions made the integration of all datasets possible, creating a collection of data inherent to urbanisation and healthcare in Sub-Saharan Africa.

### 2.3 Distribution and tier of specialisation of healthcare facilities

The spatial database of healthcare facilities (HCF) managed by the public health sector in Sub-Saharan Africa is the outcome of a recent publication (Maina et al., 2019), collecting data since 2004 in Kenya and expanding to the rest of SSA between 2012 and 2018. Data is organised in a table, which stores a series of attributes for 98,745 health facilities, including their name, type and geographic coordinates. The type is categorised by tiers of specialisation, with slight variations at national level, to indicate the technical sophistication and operational capacity of each post in terms of healthcare. Overall, tier I corresponds to primary health centres (88% of total HCF), helping local communities to face immediate needs and spread access to essential medications. Tier II facilities (10% of HCF) incorporate emergency services to deal with most diseases, injuries and immediate threats to health. Tier III structures (2% of HCF) offer more complex and specialised services, while tier IV and beyond (18 in total over all SSA, corresponding to less than 0.02%) include advanced diagnosing, training and research facilities.

### 2.4 Geographic accessibility of healthcare facilities

The accessibility map of healthcare in Sub-Saharan Africa is derived from the scientific records (Hierink et al., 2022). The data table contains a computation of population by administrative areas (GADM level 2), being able to access the nearest healthcare facility within different time thresholds (30, 60, 90, 120, 150, and 180 min). Travel speeds accounted for motorised vehicles on roads and walking off roads. Further details can be found in the cited reference (Hierink et al., 2022, para. Accessibility Model).

### 2.5 Geographic accessibility of urban centres

Another informative layer about urbanisation and infrastructure in SSA is the Global Accessibility Map (Nelson, 2008), which provides the travel time to the nearest city of at least 50 thousand inhabitants with 1 km resolution worldwide in year 2000. The friction map relies on a cost-distance model, different from the one used for healthcare facilities (Hierink et al., 2022). In this case, the definition of city is consistent with the *urban centre* according to the Degree of Urbanisation.

### 2.6 Processing method

Given the Degree of Urbanisation of the SSA territory and its 7080 subdivisions (GADM level 2) of the respective 49 countries, matching it with the underlying healthcare facilities is the next step. SSA has a population of 1.13 billion inhabitants (2020), of which 407 million live in rural areas. Processing the various datasets in a GIS environment with a common map projection allowed sampling healthcare locations on the gridded Degree of Urbanisation map and on the classified territorial units map too (Fig. 2). This led to the aggregation of healthcare locations by both gridded (GHS-SMOD) and territorial units (GHS-DUC) classification, enabling clustering and further analysis. In particular, joining the geographic accessibility of HCF (Hierink et al., 2022) to the classified territorial units map showed the urban/rural divide in terms of people's nearest health service point. In parallel, the overlay of gridded DoU (GHS-SMOD) with the population grid (GHS-POP) and the gridded Global Accessibility Map of *Urban Centres* (Nelson, 2008) revealed the infrastructural gaps making many areas in SSA isolated and disconnected from cities.

## 3 Results

### 3.1 The classification of settlements according to the Degree of Urbanisation

The majority of land surface in SSA is classified as rural, as shown in the grid map of the Degree of Urbanisation (Fig. 8c, zoom areas of the large map). Territorial units typologies are predominantly *dispersed rural areas* (class 12) and *dense towns* (class 23), by effect of which Degree of Urbanisation Grid (GHS-SMOD) class the majority of population belongs to, in each territorial unit (see the result in the DoU territorial units map in Fig. 8c).

### 3.2 Healthcare facilities by Degree of Urbanisation

**Fig. 3:**
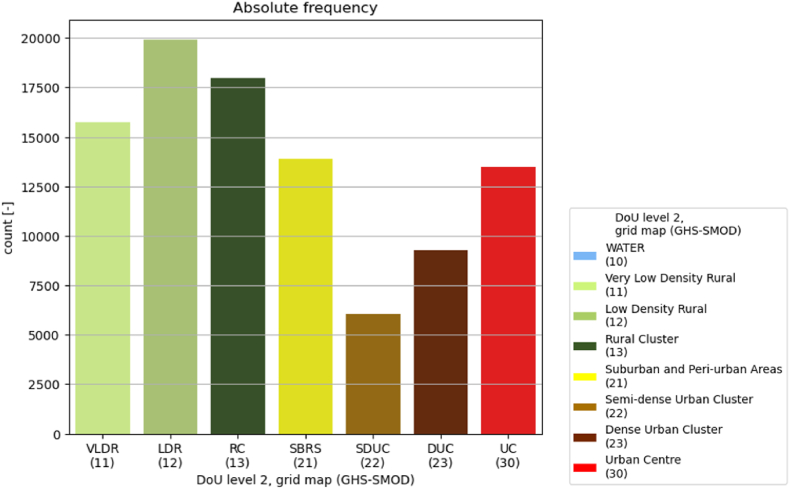
Number of HCF per DoU level 2, grid map (GHS-SMOD).

With reference to the grid map (GHS-SMOD), Fig. 3 shows how the number of healthcare facilities is higher in rural areas (classes 11, 12, 13), in particular in *low density rural* grid cells (class 12) and *rural clusters* (class 13). *Suburban/peri-urban* (class 21) and *Urban Centre* grid cells (class 30) host an almost equal number of HCF (roughly 12,500), slightly lower compared to rural areas (between 15 and 20 thousand); *dense* and *semi-dense urban clusters* (classes 23 and 22) follow with considerably lower numbers of HCF (between 5 and 10 thousand).

**Fig. 4:**
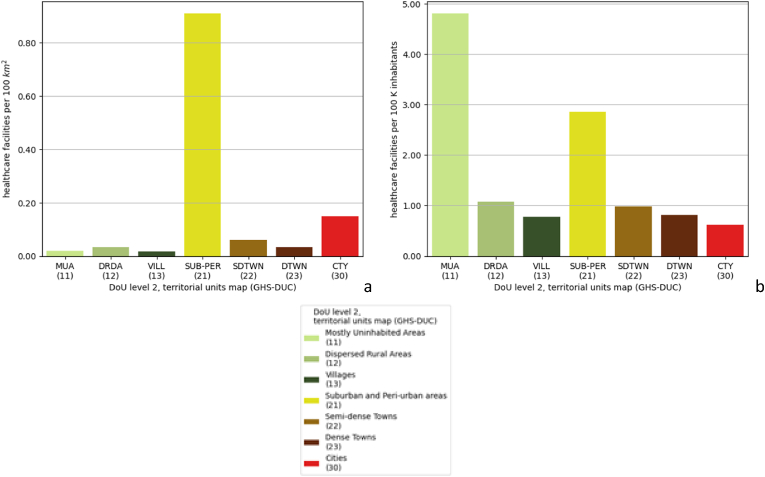
Density of HCF per (a) area and (b) population, in each class of the DoU level 2, territorial units map (GHS-DUC).

When comparing such numbers with the area and population of territorial units (Fig. 4) in the territorial units map (Fig. 8c), it emerges that urban areas (classes 30, 23, 22, 21) feature a higher number of HCF per unit of surface, in particular *suburban and peri-urban areas* (class 21), outranking *cities* by a factor of 2. Concurrently, the number of HCF per inhabitant is lower in urban areas compared with rural areas, with up to a 3.5 times difference in favour of *mostly uninhabited areas* vs *cities*.

**Fig. 5:**
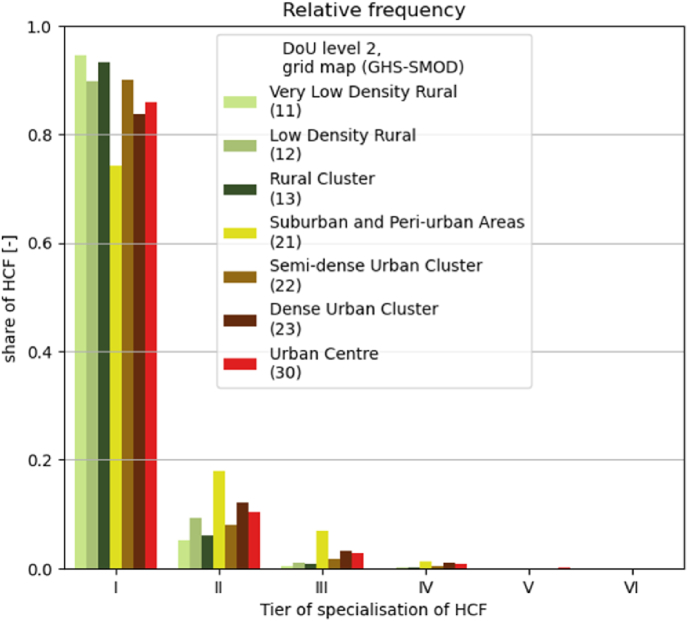
Levels of healthcare, expressed by tiers of specialisation of HCF, broken down in percentages by DoU level 2 class in the grid map (GHS-SMOD).

If the results presented so far support the logic of higher proximity in urban areas (more HCF per unit of surface) and higher capacity in rural areas (more HCF per inhabitant), the analysis of the level of healthcare shifts the balance of higher tiers of specialisation, integration and equipment to urban areas (Fig. 5).

### 3.3 Geographic accessibility of healthcare facilities by Degree of Urbanisation

**Fig. 6:**
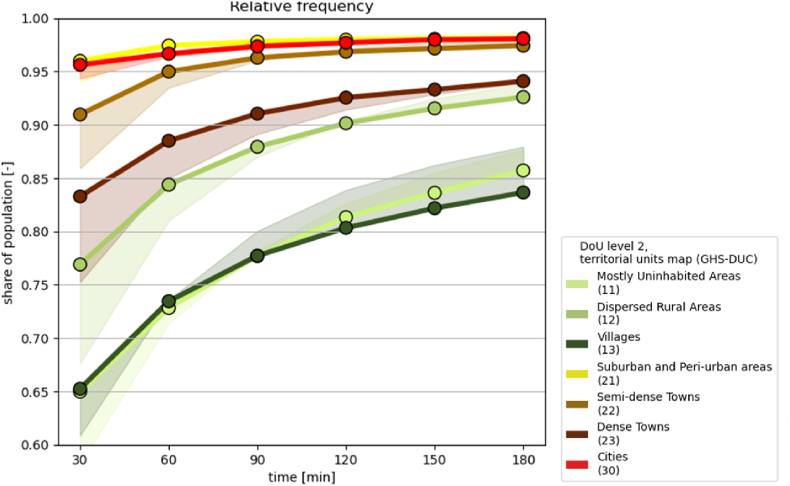
Geographic accessibility of healthcare, expressed as travel time to the nearest HCF, broken down in share of population by DoU level 2 class in the territorial units map (GHS-DUC). The coloured shade represents the difference introduced by using the constrained WorldPop dataset instead of GHS-POP for computing population.

In terms of accessibility (Fig. 6), rural areas stand out, with a 36% peak of population living more than 30 min away from the nearest healthcare centre in *mostly uninhabited areas* (class 11) and *villages* (class 13). *Cities* (class 30) and *peri-urban areas* (class 21) appear well served, with more than 95% of inhabitants living within 30 min from the nearest HCF; this share decreases to 80%–90% in *towns*. Population fractions served by nearby HCF rise more significantly over time thresholds in rural areas, with almost 10% gain between 30 min and 1 h travel time, with progressively lower amount of people being added at each 30 min timestep increase. In urban areas, the gain between 30 min and 1 h is less than 5%, progressively decreasing. Looking at the edges of the plot from the right side reveals that there is 10%–15% of people in rural areas left behind by health services, unable to reach any health post, even the lowest tier one, within 3 h time. This configures a sizable index of remoteness from healthcare (Myers et al., 2015).

This share is still between 2% and 6% in urban areas. On the contrary, the left side of the plot illustrates that more than 95% of city population could access an healthcare facility within 30 min: this share is even higher in *suburban and peri-urban areas* (class 21), but it declines for the other urban typologies of the degree of urbanisation, and namely to about 90% in *semi-dense towns* (class 22), less than 85% in *dense towns* (class 23). In the rural domain, more than 75% of the population in *mostly uninhabited areas* (class 11) reaches an healthcare facility within 30 min, with this share decreasing to about 65% of the population in *villages* (class 13) and *dispersed rural areas* (class 12).

### 3.4 Comparison across countries

**Fig. 7:**
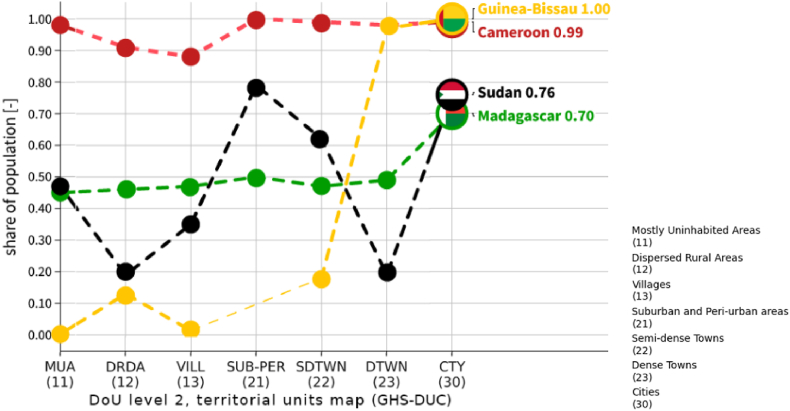
Share of population having geographic access to the nearest HCF within 30 min, by selected countries and by DoU level 2, territorial units map (GHS-DUC). Class 21 in Guinea-Bissau is missing, as there is no territorial unit classified as such.

Access to HCF does vary significantly across countries (Fig. 7), and within countries by territorial typology (Figure S. 4). Fig. 7 shows how the share of population living within 30 min of an HCF is significantly high in Cameroon (>80% of population in each class), whereas in Madagascar and Sudan it is much lower (about 40%). The figure highlights also the disparities among settlement types within the same country, as in the case of Madagascar and Sudan. The former displays a clear rural-urban gradient, whereas about 45% of the population in *mostly uninhabited areas* (class 11) has convenient access to HCF and this share increases to 70% in *cities* (class 30). In Sudan instead, there is a greater variability: in correspondence of the two peaks, about 80% of the population in *cities* (class 30) and *peri-urban areas* (class 21) has convenient access to HCF; at the two minima, in *dense towns* (class 23) and in *dispersed rural areas* (class 12), only 20%. Figure S. 4 presents data per each country, disaggregated by settlement typology, potentially informing priorities for improving access to healthcare.

## 4 Discussion

### 4.1 Healthcare is more diffused in urban areas but struggles to meet global standards

The above results show that peri-urban areas are a privileged location for healthcare facilities. The lower cost of land, combined with the proximity to main settlements and infrastructural connections to the outskirts, make peri-urban areas ideal, in particular for more specialised structures (Fig. 5). Urban areas are relatively better equipped, despite counting on one HCF per every 20 thousand inhabitants in cities and one per every 10 thousand inhabitants in towns, which means one HCF in 100 km^2^ of cities and one in 200 km^2^ of towns. Rural areas though, count on less than one HCF in 400 km^2^, and even if there is roughly one HCF per every 6 thousand inhabitants, it may be too distant, as between 15% and 30% of rural population lives more than 1 h away from the closest health point. To meet the minimum of 18 beds per every 10 thousand inhabitants recommended under SDG 3.8 (United Nations Human Settlements Programme (UN-Habitat) & United Nations Statistics Division (UNSD), 2023), the current HCF in SSA should host between 10 and 36 beds each, which is unlikely especially for Tier I structures.

### 4.2 Contrasting healthcare standards: Comparing Europe and Sub-Saharan Africa

These figures become more informative when compared to Europe (Figure S. 3), where similar surface densities of HCF are observed (one in 400 km^2^ in rural areas vs one in 200 km^2^ in towns and one in 50 km^2^ in cities). Concurrently, Europeans benefit from the presence of 3–6 HCF per every 100 thousand inhabitants in rural areas, 3 to 4 in urban areas: such HCF grant a capacity of 45 beds per every 10 thousand inhabitants, based on available country data (Geographical information system of the Commission (GISCO) & Eurostat, 2020). Despite seemingly lower, European figures may be comparable to the number of HCF belonging at least to Tier II, in SSA: in this case, the capacity is circa 3 times higher in Europe, considering the number of HCF per inhabitant, except for peri-urban areas and low-density rural areas. Still, upper tiers of healthcare in Europe respond to higher standards compared to SSA, whose capacity may be overestimated in this context.

### 4.3 A growing population increases the need for infrastructure and healthcare

The considerations above indicate a relatively dense distribution of HCF on the SSA territory; instead, the low geographic accessibility resulting from the lack of adequate infrastructure to reach the health points, especially in rural areas, can be a serious challenge. More than 400 million people in SSA live beyond 3 h away from the nearest urban centre, of which almost 250 million are settled in the rural domain (Fig. 9). In such conditions, many countries in SSA struggle to provide prompt access to healthcare, within 30 min (Figure S. 4).

**Fig. 8:**
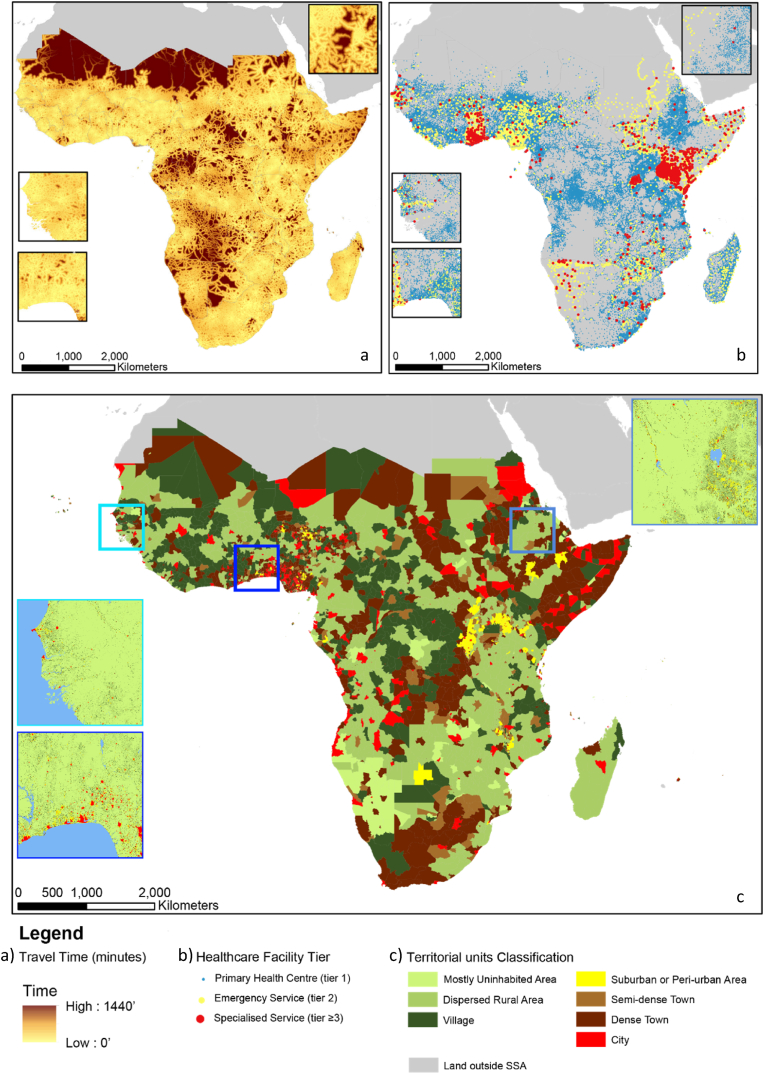
(a) Geographic accessibility of Urban Centres (settlement with more than 50 thousand inhabitants) (Nelson, 2008); (b) map of healthcare facilities in Sub-Saharan Africa, by tier of specialisation (Maina et al., 2019). (c) Degree of Urbanisation level 2 classification at territorial units level (GHS-DUC) and at grid level (GHS-SMOD), in zoom squares. Cape Verde is omitted in sub-figures a) and b).

**Fig. 9:**
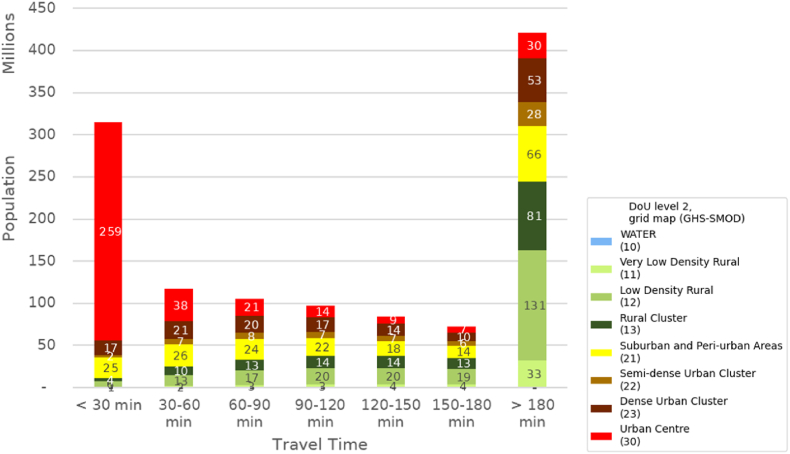
Amount of population in each travel time class of geographic accessibility to the nearest urban centre, by DoU Level 2 class in the grid map (GHS-SMOD).

According to recent forecasts (DG REGIO, 2020), 17,140 km^2^ of new built-up surface is going to be constructed in SSA between 2020 and 2030. If, in relation with the population increase, there will be a need for 6200 new HCF in the region by 2030, to meet the international standards (Falchetta et al., 2020), it means that a new health point should be placed per each 2.76 km^2^ increase in the built-up surface. In other words, every new district being developed should include its own healthcare facility, with particular attention to rural areas.

### 4.4 Limitations of the study

The analyses conducted here led to these preliminary results, employing an agreed definition of urbanisation, consistent across national boundaries, which allows for international comparisons, unprecedented in this domain. The source data referenced in peer-reviewed publications are the most complete to date and constitute the basis of the present work, but may not be exhaustive. Among the limitations of the study, the completeness of the HCF dataset is noteworthy: in fact, private HCF, which may extend the population coverage, are not included in the source dataset. Another aspect is inherent to the capacity of each HCF in terms of patients, which the cited source data do not qualify. This characteristic may vary considerably. The definition of tiers of HCF and the effective quality of services may also differ a lot among countries in SSA, undermining the reliability of specific conclusions. However, analysis of access by actual quality of service is paramount and deserves further research. Moreover, it should be noted that estimating potential and generalized geographic access is different from assessing effective access to healthcare. The mere presence and geographical access to a health point does not necessarily mean receiving care, nor appropriate and quality treatments, as other factors become relevant. However, having geographic access is generally a necessary condition (although not sufficient) to receiving healthcare.

The accessibility estimation is limited by the assumptions of the healthcare friction map (Hierink et al., 2022), which does not take slope into account nor other travel impediments, such as conflict areas. Within cities, travel time may also be increased due to traffic conditions and other urban barriers^5^ (Banke-Thomas et al., 2021), which are not considered in the model. These factors may cause overestimation of rates of geographic access to healthcare: further details can be found in the discussion section of the reference paper (Hierink et al., 2022). In addition, with reference to Fig. 9, the accessibility of urban centres is calculated differently from the one of healthcare facilities. Another level of uncertainty for the accessibility maps resides in the population estimates, which rely on the GHS-POP grids (Schiavina, Marcello, Freire, Sergio, et al., 2022). In previous work (Hierink et al., 2022), the utilization of distinct population grids for assessing healthcare accessibility in Sub-Saharan Africa revealed minor variances when utilizing the GHS-POP dataset in comparison to the WorldPop restricted dataset. This was observed in both the disaggregation of population by travel time to the nearest HCF (see shaded curves in Fig. 6) and in terms of urban/rural classification (Beale, 2022). Therefore, GHS-POP constitutes the benchmark of the current work, for uniformity across all datasets. Regarding the Degree of Urbanisation, the literature includes a discussion on its main limitations (Dijkstra et al., 2020): in particular, the size of the territorial units has an impact on their classification by population majority rule.

## 5 Conclusions

The Degree of Urbanisation method introduces the possibility to monitor the urban-rural continuum in access to resources and services across countries and continents, thanks to a common international definition. Sub-Saharan Africa has been historically lacking healthcare, while its population and demographic and land urbanisation continue to grow.

This work characterizes distribution and accessibility of HCF by Degree of Urbanisation Level 2 for the first time. Results suggest that, despite the capacity in terms of HCF per inhabitant being at least three times lower than in Europe, SSA follows a common trend of urban-rural gradient in the quantity of HCF. Rural areas rely on two to three times more HCF per inhabitant compared to urban areas, but they are spread on a much larger territory. This makes it difficult to reach them, with circa three times more population in rural areas struggling to access the nearest health point in less than 1 h, compared to cities. In addition, rural areas are equipped with less specialised structures compared to urban areas. Reliable and adequate infrastructure, even before the placement of new healthcare facilities, is a crucial need.

The Degree of Urbanisation delineates and classifies settlements regardless of the presence of facilities, making it ideally suited to assess and plan the provision of services such as healthcare.

The post-pandemic phase of COVID-19 constitutes a precious occasion for designing new, accessible spaces that cover people's basic needs, including health wise (United Nations Human Settlements Programme (UN-Habitat), 2021). The outcomes of this research constitute one contribution for identifying under-served populations in SSA and act accordingly.

## Author statement

The designations employed and the presentation of materials and maps do not imply the expression of any opinion whatsoever on the part of the European Union concerning the legal status of any country, territory or area or of its authorities, or concerning the delimitation of its frontiers or boundaries that if shown on the maps are only indicative. The boundaries and names shown on maps do not imply official endorsement or acceptance by the European Union. The views expressed herein are those of the authors and do not necessarily reflect the views of the European Union. Authors declare no conflict of interest.

## Footnotes

1 https://www.who.int/data/GIS.

2 https://www.who.int/news/item/10-03-2022-who-global-health-facilities-database-ensuring-access-to-primary-healthcare-and-uhc.

3 https://ghsl.jrc.ec.europa.eu/degurba.php.

4 https://data.worldbank.org/country/ZG.

5 The website https://www.ontimeconsortium.org/contains useful references to this issue, concerning the accessibility of pregnancy and maternity healthcare facilities.
