# Supplementary material for: Estimating geographic access to healthcare facilities in Sub-Saharan Africa by Degree of Urbanisation
Source: Appl Geogr. 2023 Nov;160:None. doi: 10.1016/j.apgeog.2023.103118 (PMC10630936; doi:10.1016/j.apgeog.2023.103118)
Supplement: Multimedia component 1 [file mmc1.docx]

# Supplementary material

From Figure 4 (left), it is clear that rural areas benefit from less HCF per km^2^ compared to urban areas. However, with an increasing population density, the density of HCF per km^2^ grows more in rural areas than in urban ones (Figure S. 1). This means that rural units within the population density range of 10-100 inhabitants / km^2^, often host several HCF.


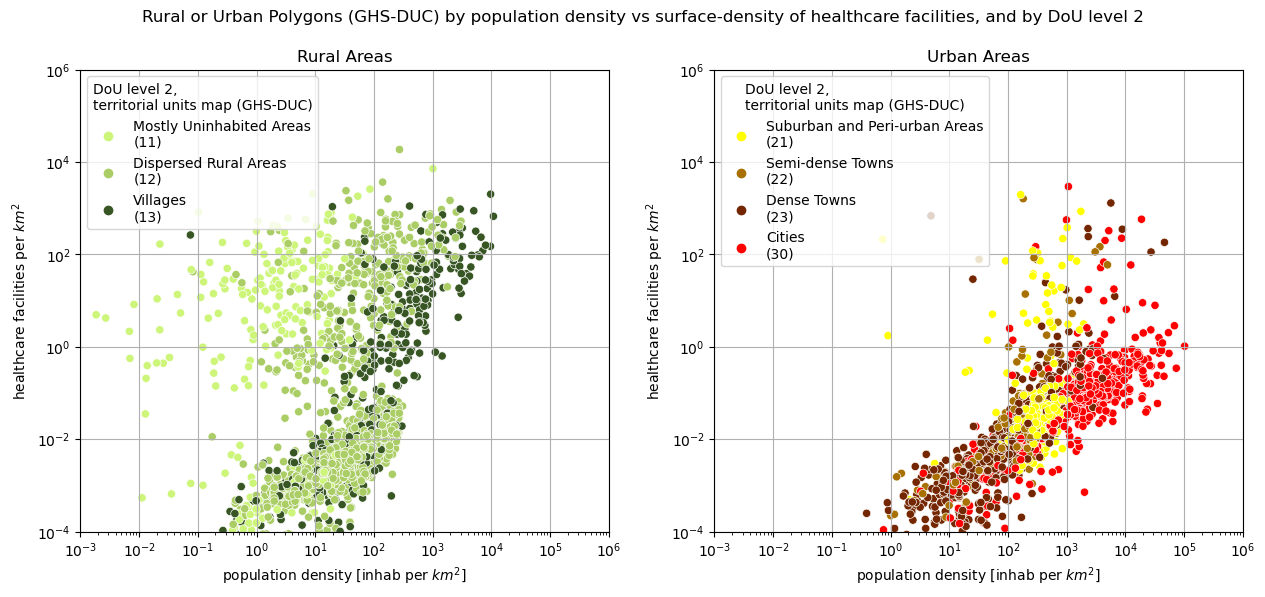


b

a

Figure S. 1: Scatterplots of territorial units representing surface density of HCF as a function of population density (axes in logarithmic scale), by (a) rural or (b) urban classes in DoU level 2, territorial units map (GHS-DUC).

In Figure 6, *dense towns* (class 23) and *villages* (class 13) have lower accessibility to HCF than the rest of urban and rural classes respectively. This may be an effect of several factors. First, the areal breakdown of territorial units in DoU grid classes: due to population majority rule, territorial units classified as *dense towns* or *villages* are often composed by large rural areas, larger than in other classes (Figure S. 2a). Secondly, being intermediate classes, *villages* and *dense towns* feature the lowest combination of HCF per inhabitant and HCF per km^2^ (Figure S. 2b).


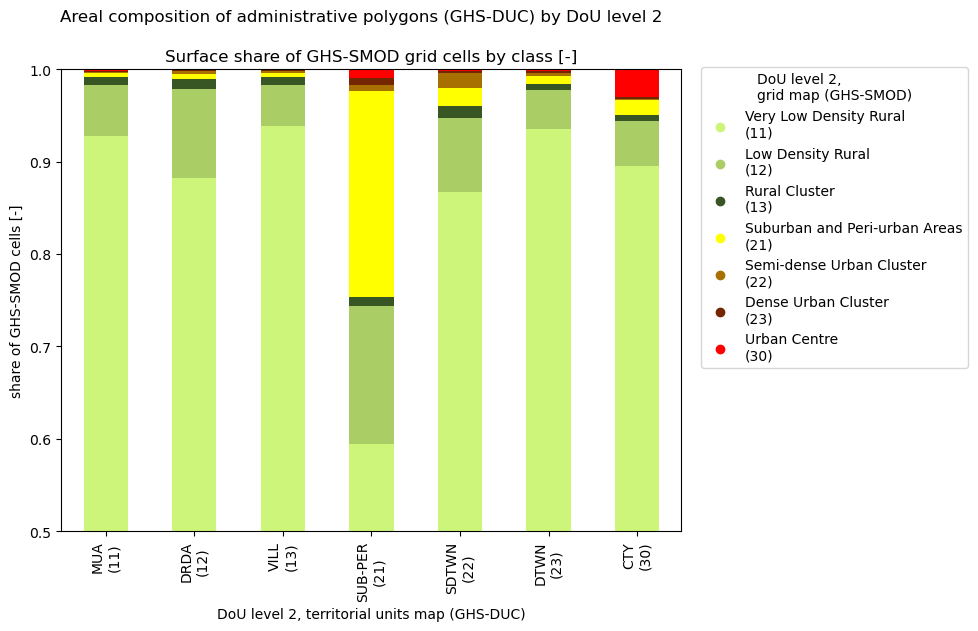


a


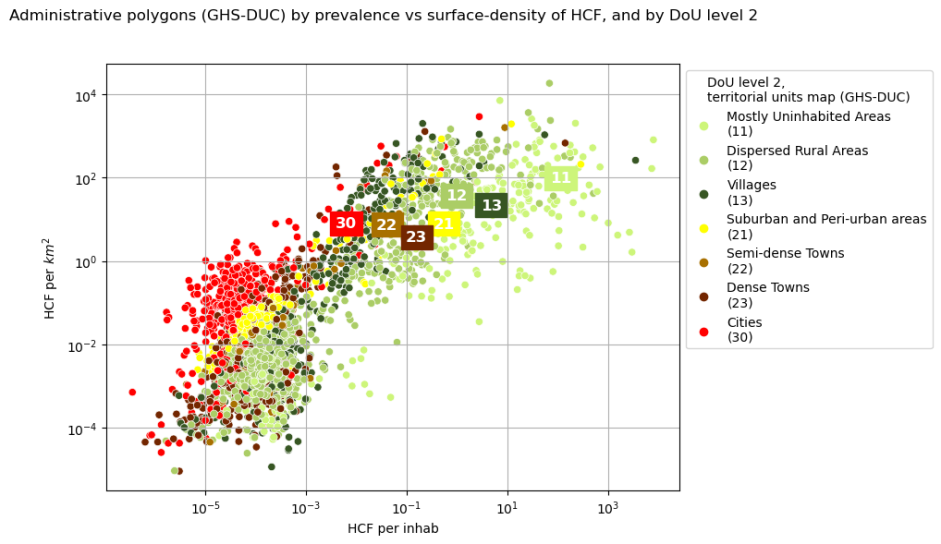


b

Figure S. 2: (a) Areal composition of territorial units in DoU level 2 classes, grid level map (GHS-SMOD), as a share of the cumulative area by DoU level 2, territorial units map (GHS-DUC). (b) Scatterplot of territorial units by number of HCF per inhabitant as a function of surface density of HCF per km^2^ (axes in logarithmic scale), coloured by DoU level 2 classes, territorial units map (GHS-DUC), bottom. Centroids (means of x and y values) of point clouds from DoU level 2 classes are represented as labelled rectangles.

The presented figures of HCF per km^2^ and per inhabitant in SSA may be more informative when compared to other regions of the world. European figures may be comparable to the number of HCF belonging at least to Tier II, in SSA, to neglect primary health centres that in most cases constitute simple drugs and pharmaceutical dispatch facilities (Maina et al., 2019, tbl. 2). The density plots for European countries (EU27) show a much different situation (Figure S. 3), especially in the number of HCF per inhabitant (Figure S. 3, right), but similar trends between urban and rural areas.

**Europe**


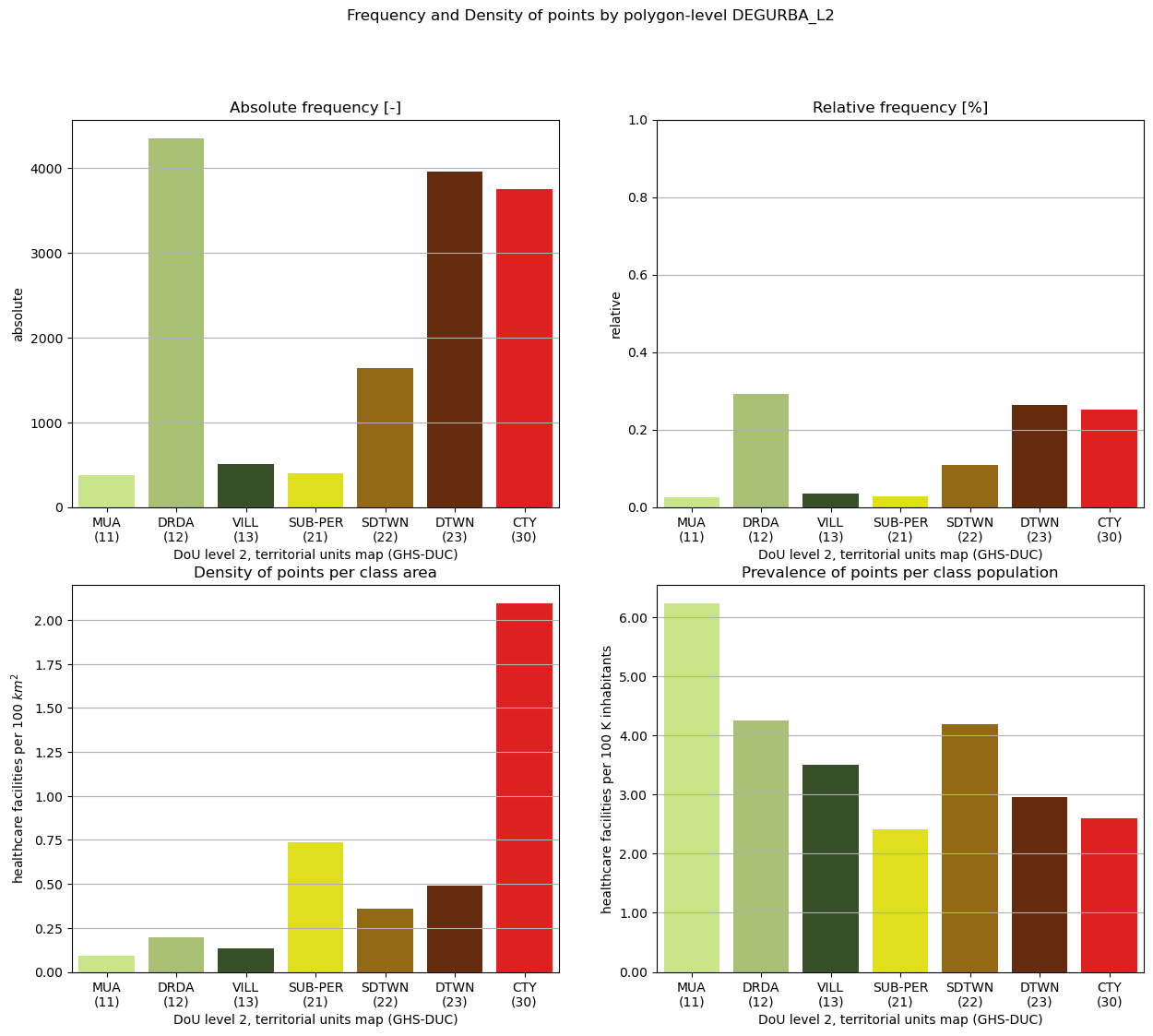

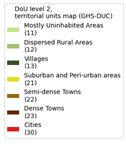


a

**SSA (Tiers II and up, to match the European standard)**


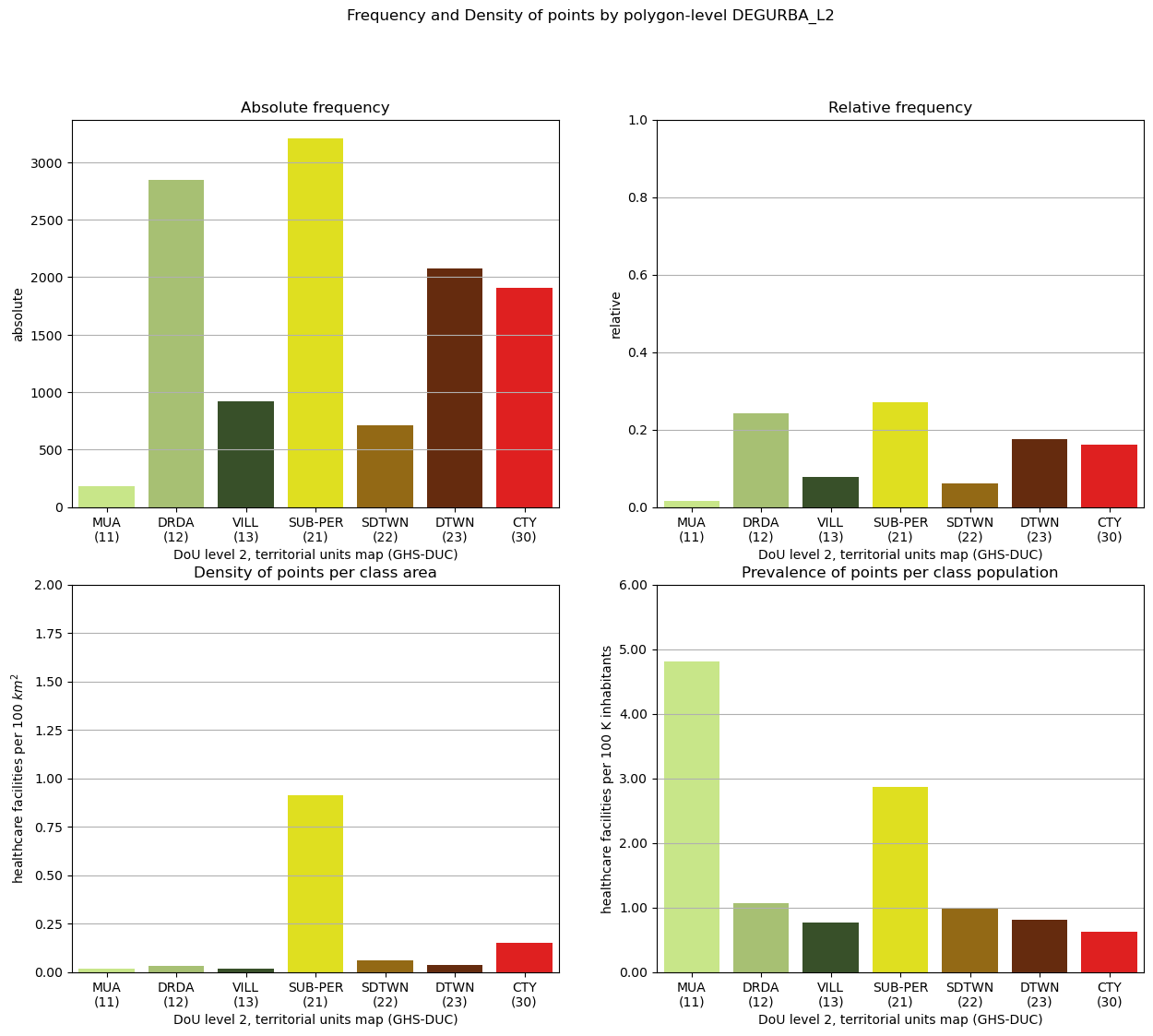

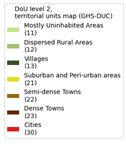


b

Figure S. 3: (a) Europe EU27, and (b) Sub-Saharan Africa: density of HCF per area (left) and population (right), in each class of the DoU level 2, territorial units map (GHS-DUC).


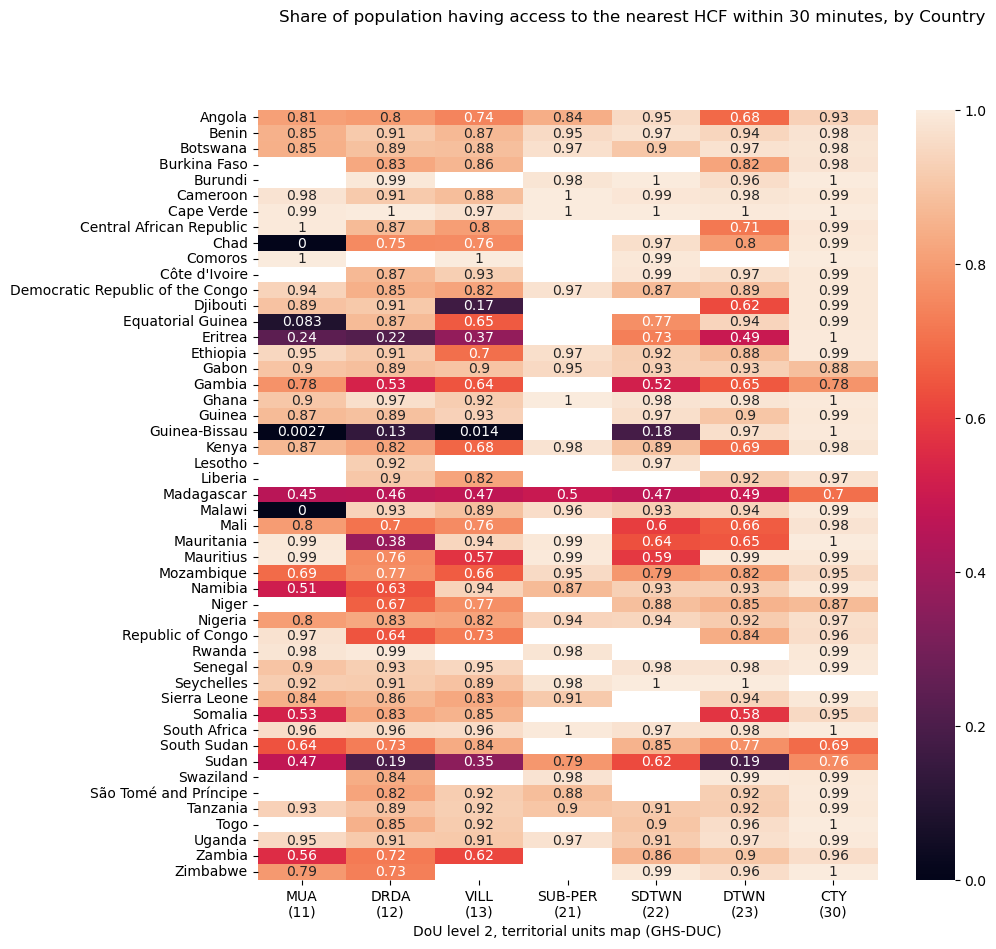

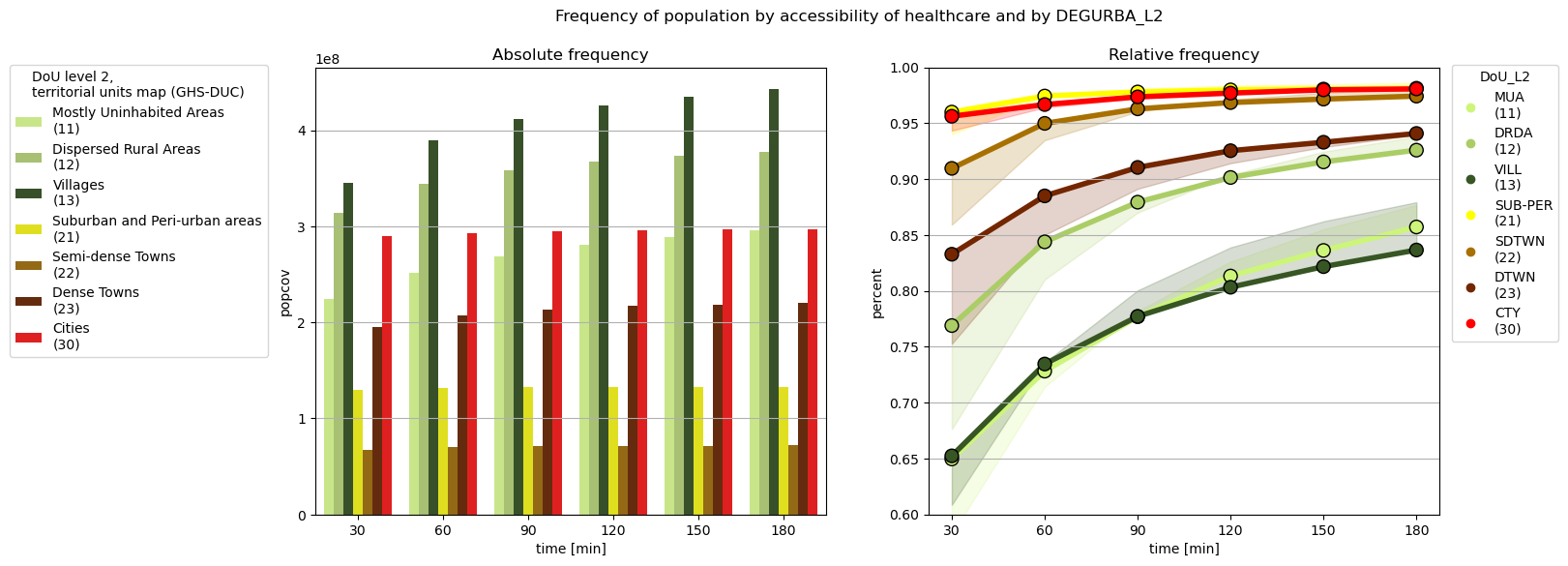


Figure S. 4: Share of population having geographic access to the nearest HCF within 30 minutes, by country and by DoU level 2, territorial units map (GHS-DUC).
